# Feasibility of “cold” triangle robotic pancreatoduodenectomy

**DOI:** 10.1007/s00464-022-09411-7

**Published:** 2022-07-26

**Authors:** Emanuele F. Kauffmann, Niccolò Napoli, Michael Ginesini, Cesare Gianfaldoni, Fabio Asta, Alice Salamone, Gabriella Amorese, Fabio Vistoli, Ugo Boggi

**Affiliations:** 1 Division of General and Transplant Surgery, University of Pisa, Via Paradisa 2, 56124 Pisa, Italy; 2 Division of Anesthesia and Intensive Care, Azienda Ospedaliero Universitaria Pisana, Pisa, Italy

**Keywords:** Pancreatoduodenectomy, Pancreatic cancer, Triangle, Mesopancreas, Robotic, Robot assisted

## Abstract

**Background:**

Triangle pancreatoduodenectomy adds to the conventional procedure the en bloc removal of the retroperitoneal lympho-neural tissue included in the triangular area bounded by the common hepatic artery (CHA), the superior mesenteric artery (SMA), and the superior mesenteric vein/portal vein. We herein aim to show the feasibility of “cold” triangle robotic pancreaticoduodenectomy (C-Tr-RPD) for pancreatic cancer (PDAC).

**Methods:**

Cold dissection corresponds to sharp arterial divestment performed using only the tips of robotic scissors. After division of the gastroduodenal artery, triangle dissection begins by lateral-to-medial divestment of the CHA and anterior-to-posterior clearance of the right side of the celiac trunk. Next, after a wide Kocher maneuver, the origin of the SMA, and the celiac trunk are identified. After mobilization of the first jejunal loop and attached mesentery, the SMA is identified at the level of the first jejunal vein and is divested along the right margin working in a distal-to-proximal direction. Vein resection and reconstruction can be performed as required.

C-Tr-RPD was considered feasible if triangle dissection was successfully completed without conversion to open surgery or need to use energy devices. Postoperative complications and pathology results are presented in detail.

**Results:**

One hundred twenty-seven consecutive C-Tr-RPDs were successfully performed. There were three conversions to open surgery (2.3%), because of pneumoperitoneum intolerance (*n* = 2) and difficult digestive reconstruction. Thirty-four patients (26.7%) required associated vascular procedures. No pseudoaneurysm of the gastroduodenal artery was observed. Twenty-eight patients (22.0%) developed severe postoperative complications (≥ grade III). Overall 90-day mortality was 7.1%, declining to 2.3% after completion of the learning curve. The median number of examined lymph nodes was 42 (33–51). The rate of R1 resection (7 margins < 1 mm) was 44.1%.

**Conclusion:**

C-Tr-RPD is feasible, carries a risk of surgical complications commensurate to the magnitude of the procedure, and improves staging of PDAC.

**Graphical abstract:**

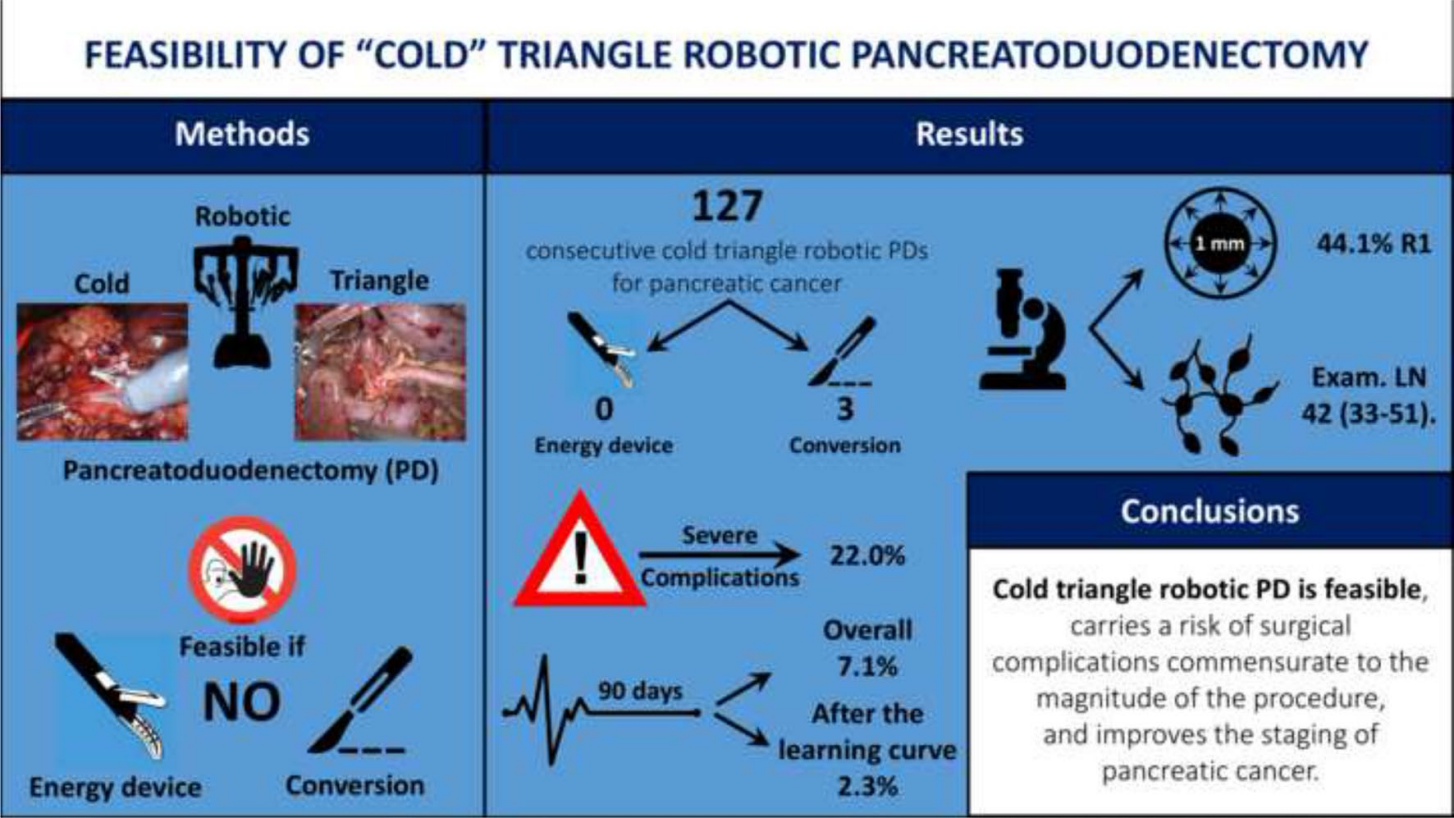

**Supplementary Information:**

The online version contains supplementary material available at 10.1007/s00464-022-09411-7.

Pancreatic ductal adenocarcinoma (PDAC) has no early stage, because of the unfavorable biology characterized by early hematogenous spread, retroperitoneal and perineural invasion, and high rate of lymph node metastasis [1]. This is the main reason why all efforts to improve survival by means of extended surgery alone have failed [2]. However, survival in resected PDAC is clearly influenced by surgery-related factors, such as number of retrieved lymph nodes [3, 4], margin status [5], and occurrence of postoperative complications [6]. Therefore, high-quality surgery remains key to preserve the few individual chances of long-term survival in resected PDAC.

In 2012 Adham described the presence of a triangular flap of tissues extending from the posterior surface of the head of the pancreas, behind the superior mesenteric/portal vein (SMV/PV), to the celiac trunk (CT) and the superior mesenteric artery (SMA) and proposed a technique for en bloc radical removal of these tissues during pancreatoduodenectomy (PD) [7]. On practical grounds, the triangle space corresponds to the extrapancreatic nerve plexus (ExNP), containing also lymphatic channels and lymph nodes that was extensively studied by Japanese authors [8, 9]. In 2019, the Heidelberg group named triangle PD the en bloc removal of ExNP during PD and underlined how this procedure is a “*vessel-oriented pancreatic head resection”* [10]. Considering that approximately 20% of the patients develop local-only recurrence after PD for PDAC [11] and that the site of recurrence typically corresponds to the “triangle” [12], triangle PD could reduce the incidence of local recurrence following PD for PDAC.

Our group performs triangle PD for PDAC since the early 2000’s [13], taking inspiration from the Japanese studies on ExNP involvement in resectable PDAC [8, 9]. As suggested by some groups [10, 14], in open PD we prefer to pursue sharp (i.e., “cold”) periadvential dissection of large peripancreatic arteries (also named arterial divestment).

Energized dissection has clearly facilitated and expedited many surgical procedures, especially in minimally invasive surgery. However, the use of energy devices is associated with the intrinsic risk of proximity tissue damage [15]. In minimally invasive PD, sharp dissection is considered time consuming and could increase the risk of intraoperative bleeding.

In this article we aim to demonstrate the feasibility of cold robotic triangle PD (C-Tr-RPD). Cold dissection means sharp arterial divestment using only the tip of robotic scissors, without energy devices.

## Materials and methods

A retrospective analysis of a prospectively maintained database was performed for all C-Tr-RPDs performed for PDAC and malignant intraductal papillary mucinous neoplasms (IPMNs) at the Division of General and Transplant Surgery of the University of Pisa between August 1st, 2009 and September 30th, 2021.

This study was approved by the Institutional Ethical Board of the University of Pisa and was performed according to the principles of the Declaration of Helsinki [16] and the Strengthening the Reporting of Observational studies in Epidemiology (STROBE) guidelines on reporting on observational studies [17].

Categorical variables are presented as rates and proportions. Continuous variables are reported as mean ± SD if normally distributed or as median and interquartile range (IQR) if not.

### Study design

This study aims to show feasibility of C-Tr-RPD. The main study endpoint was therefore a composite index made by conversion to open surgery due to inability to complete triangle dissection or need to use energy devices to do so (i.e., harmonic shears and/or radiofrequency or microwave-powered devices).

Secondary study endpoints were incidence and severity of postoperative complications [18], post-pancreatectomy hemorrhage (PPH) [19], delayed gastric emptying (DGE) [20], and chyle leak [21]. Postoperative complications ≥ grade III, according to Clavien–Dindo [18], were considered severe complications. The cumulative burden of postoperative complications was estimated using the comprehensive complication index (CCI) [22].

The following parameters were also recorded: operative time, pylorus preservation, need and type of vascular resection [23], length of hospital stay, 90-day hospital readmission, 90-day mortality, 90-day mortality following completion of the learning curve [24], reoperation, and interventional procedures.

All specimens were analyzed according to the LEEPP protocol [25], as previously reported in detail [26]. Seven margins were assessed: anterior surface, posterior surface, vein bed, SMA groove, pancreatic neck, proximal duodenum/stomach, and common bile duct. Margins were defined positive (R1) if tumor cells were detected ≤ 1 mm of any margin.

Additional pathology data included tumor type (i.e., PDAC or malignant IPMN), tumor size, T status, N status, presence of perineural infiltration, number of examined lymph nodes, and number of metastatic lymph nodes.

### Selection criteria

Selection criteria for robotic PD (RPD) for PDAC gradually evolved over time [27, 28]. Currently, we still do not accept patients with extremely high body mass index (i.e., ≥ 35 kg/m^2^ for males and ≥ 40 Hg/m^2^ for females), patients with narrowing/occlusion of the SMV/PV causing portal hypertension, and patients with locally advanced tumors. Occasionally, patients with intraoperative evidence of limited arterial involvement were managed robotically [29].

### Surgical technique

Only triangle dissection is described here. Details on other operative steps of RPD are available from our previous articles [27, 28]. For all procedures a da Vinci Surgical System (Intuitive Surgical, Sunnyvale, CA, USA) was used. A da Vinci Si System was used in 72 C-Tr-RPDs (56.7%), while a da Vinci Xi System was used in the remaining 55 C-Tr-RPDs (43.3%). For the purpose of this description there is no difference between the two da Vinci Systems.

Triangle dissection is divided in four main steps for the purpose of clarity. Actual operative workflow may see some overlap of these steps, based on individual anatomy and tumor characteristics.

### General principles

**Fig. 1:**
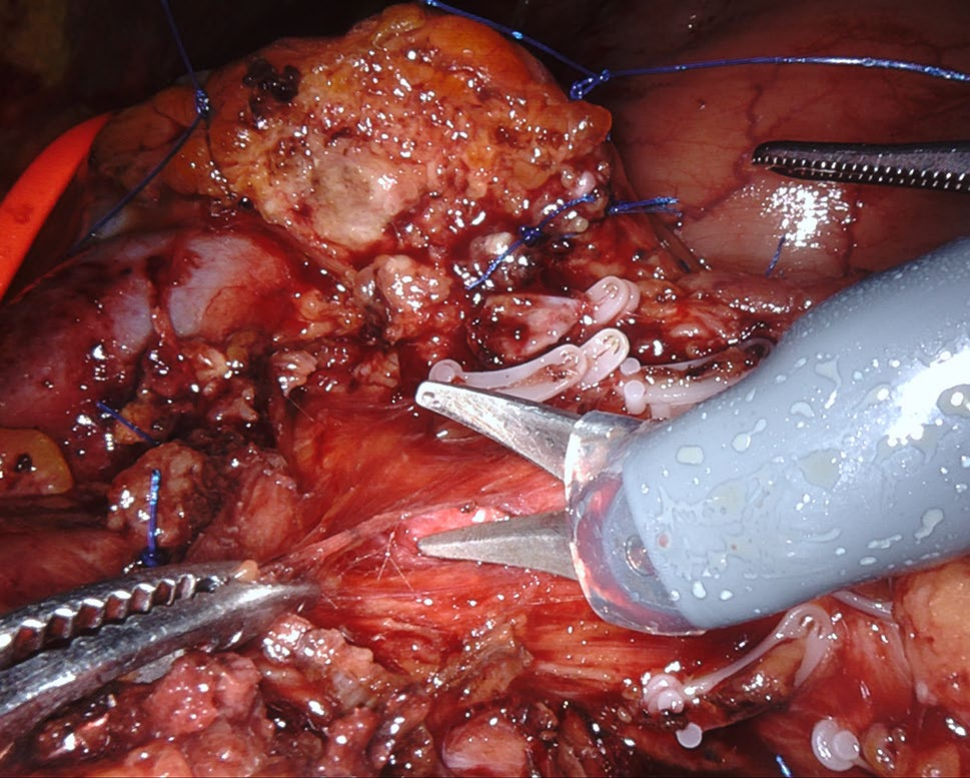
Arterial divestment by cold dissection (tip of robotic scissors)

As already stated, dissection around large visceral arteries and SMV/PV is performed using only the tip of robotic scissors (Fig. 1). Bleeding sites are fixed selectively by fine sutures (6/0 or 5/0 polypropylene), ligatures (3/0 or 2/0 linen), or robotic Hem-o-lok clips. Hem-o-lok clips can be applied by the assistant at the table, but we prefer to use the dedicated robotic instrument, because the endowrist® mechanism permits precise positioning of each clip even in deep and narrow spaces with an ideal alignment. Robotic Hem-o-lok clips are also used to seal large lymphatic channels. Considering the vessel-oriented approach of this procedure and the mechanical sealing of most dissected tissues, there is limited need for additional hemostasis. When required, small bleeders are fixed by selective use of bipolar energy. A set of laparoscopic bulldog clamps are immediately available for temporary control of major bleeding from large vessels.

Presence of variations in arterial liver supply, number and position of large pancreatoduodenal arteries, and origin of dorsal pancreatic artery are carefully noted on preoperative computed tomography scan. Branching of SMA and superior mesenteric vein is also noted [30].

#### Step 1

**Fig. 2:**
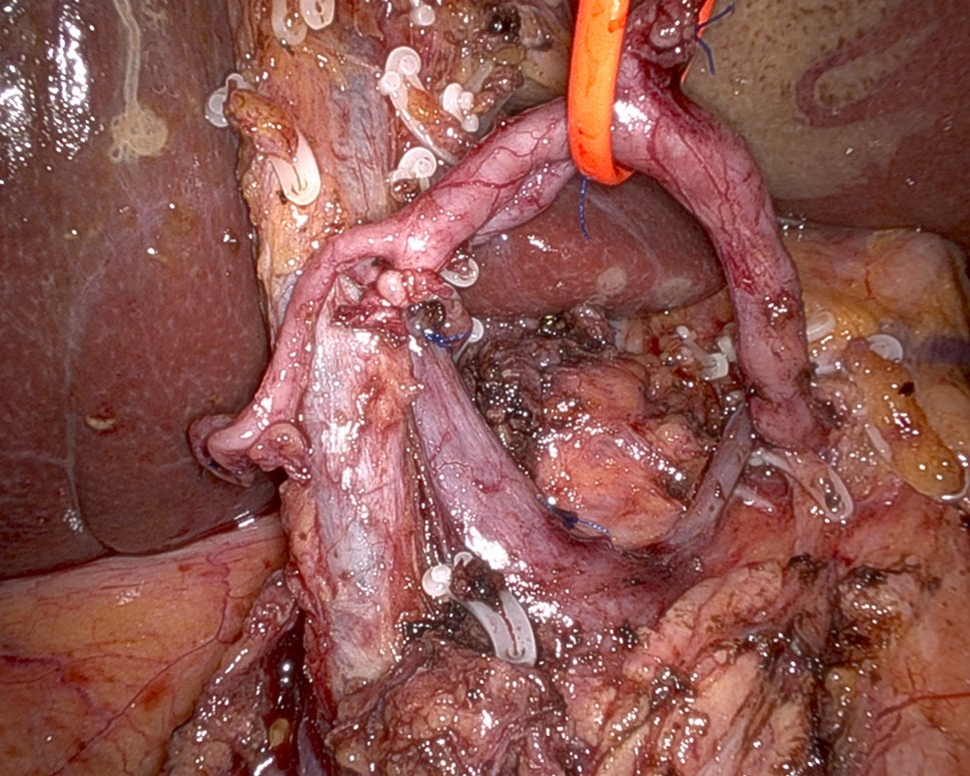
Circumferential clearance of common hepatic artery

**Fig. 3:**
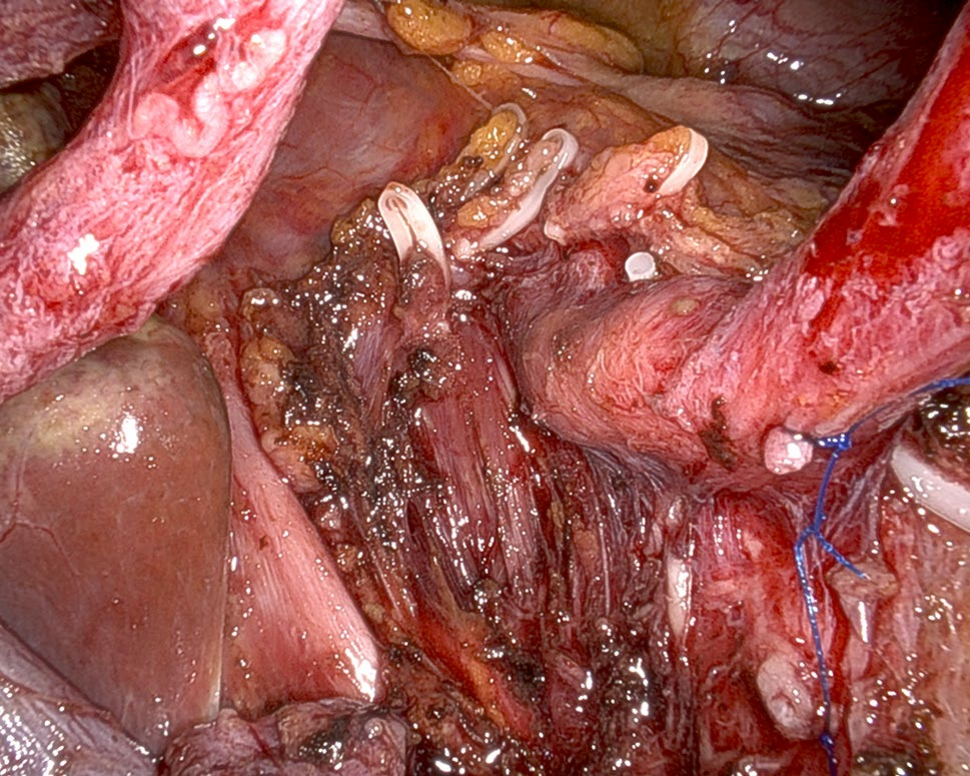
Descending dissection along the right side of the celiac trunk reaching the right diaphragmatic crus

After descending lymphadenectomy of the hepatoduodenal ligament (lymph node station n. 12), clearance of the triangle begins by divesting the common hepatic artery, working in a lateral-to-medial direction. After division between ligatures of the gastroduodenal artery, the common hepatic artery is encircled with a vessel loop for gentle handling during circumferential clearance (lymph node stations n. 8a and 8p) (Fig. 2). Once the CT is reached, dissection continues on a descending direction until the right diaphragmatic crus is reached (lymph node station n. 9) (Fig. 3) (video 1).

#### Step 2

**Fig. 4:**
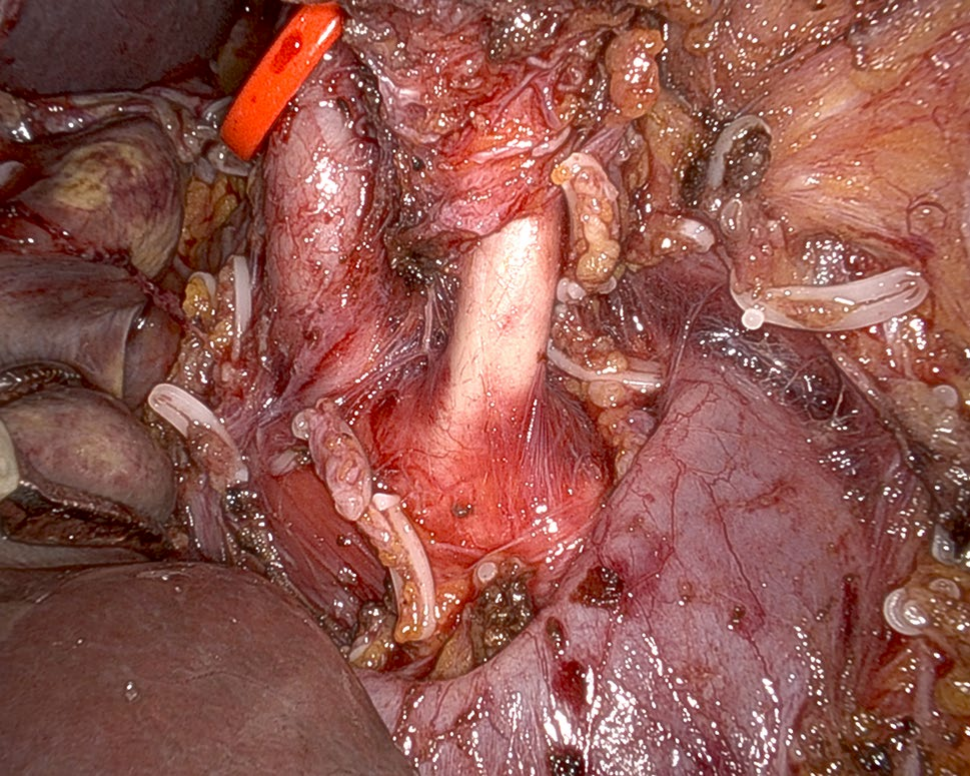
Working from a posterior approach and following centripetal clearance of retroperitoneal lympho-neural tissues, the origin of the superior mesenteric artery and celiac trunk are clearly identified

Step 2 begins with a wide Kocher maneuver to allow free access to the origin of SMA and CT. Lympho-neural tissues above the left renal vein and medial to the inferior vena cava are dissected in a centripetal direction to clearly expose the SMA and the CT. The right celiac ganglion is also usually removed en bloc with surrounding retroperitoneal tissues (lymph node station n. 16a2). Origins of SMA and CT are identified for safety purposes and to provide a clear line of dissection during divestment of the right side of the SMA (Fig. 4) (video 2).

#### Step 3

**Fig. 5:**
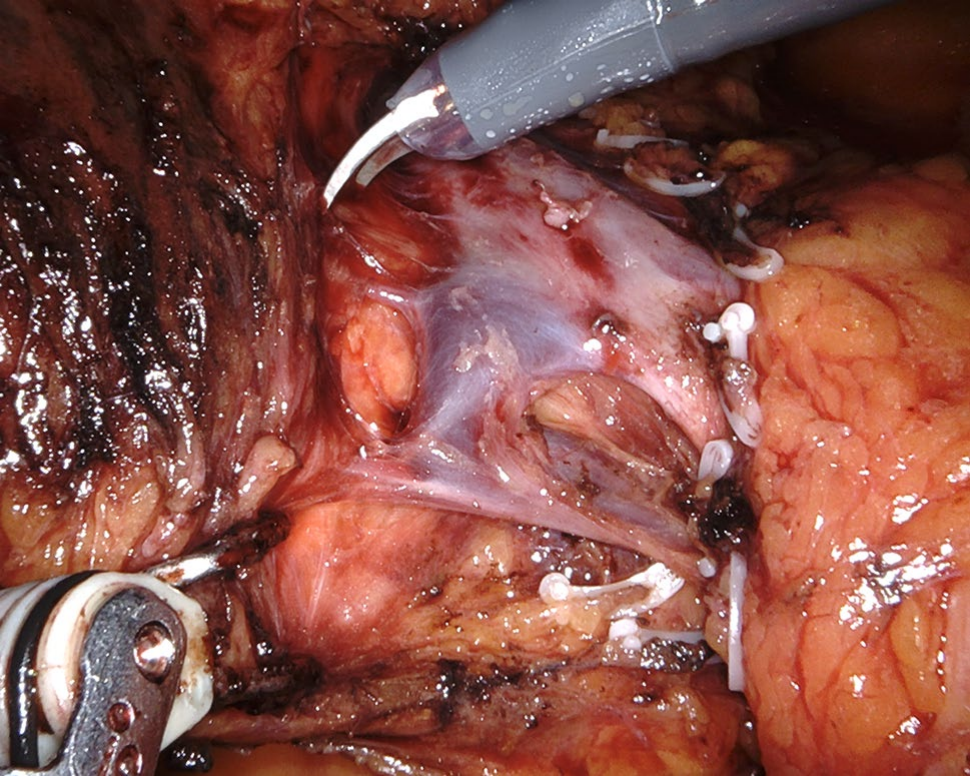
The superior mesenteric artery runs just posterior to the first jejunal vein

The peritoneum behind the third duodenal portion is opened and the first jejunal loop is mobilized to the right side of mesenteric vessels. With the forth robotic arm hanging the duodenum to the right side, the jejunal mesentery is divided using harmonic shears. Especially for tumors located in the uncinated process it is important to remove the mesentery of the first jejunal loop (lymph node station n. 14d) that requires the sacrifice of one or two jejunal branches. The first jejunal vein can be used as a landmark for safe location of the SMA considering that it runs dorsal to the SMA in 63.0–87.2% of the patients [31]. Once the SMA is identified, dissection proceeds proximally along the periadvential plane (lymph node stations n. 14a and 14 b) (Fig. 5) (video 3).

#### Step 4

**Fig. 6:**
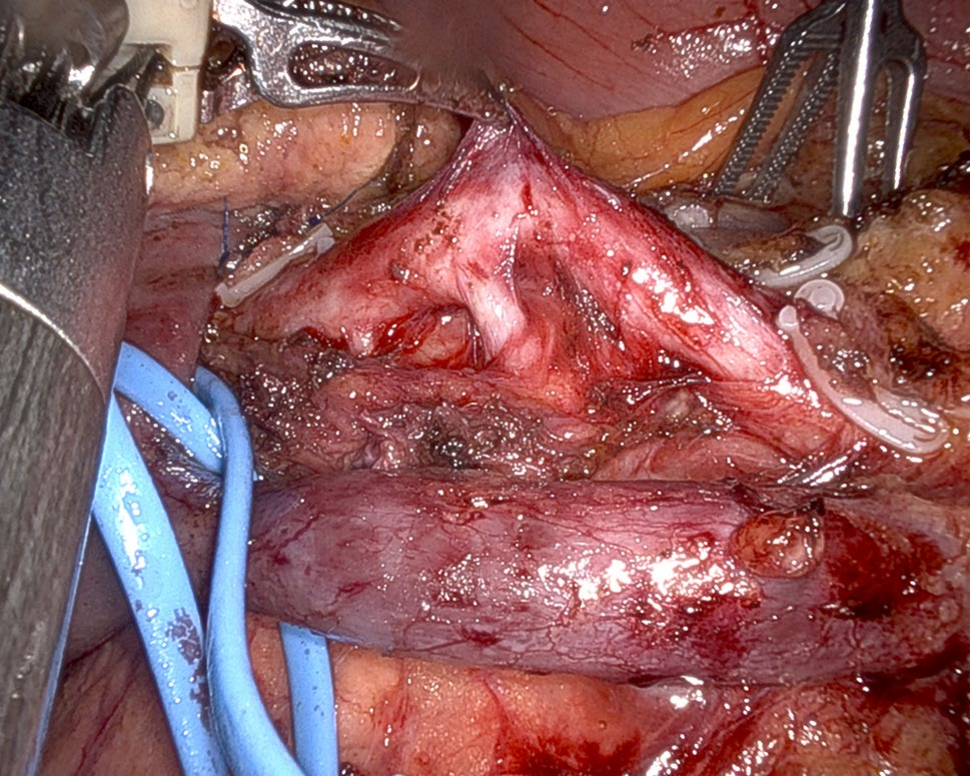
Working from an anterior approach the right side of the superior mesenteric artery is divested. Note two pancreatoduodenal arteries that are clearly identified in preparation for selective ligature and division

**Fig. 7:**
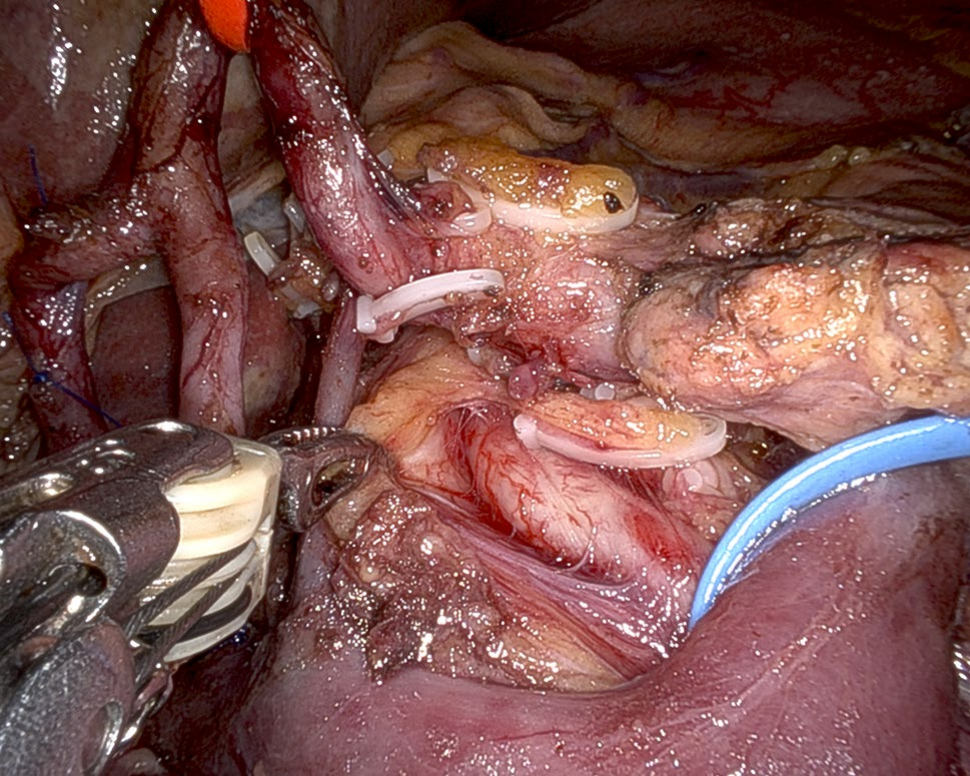
Retroperitoneal lympho-neural tissues are peeled off the superior mesenteric artery

**Fig. 8:**
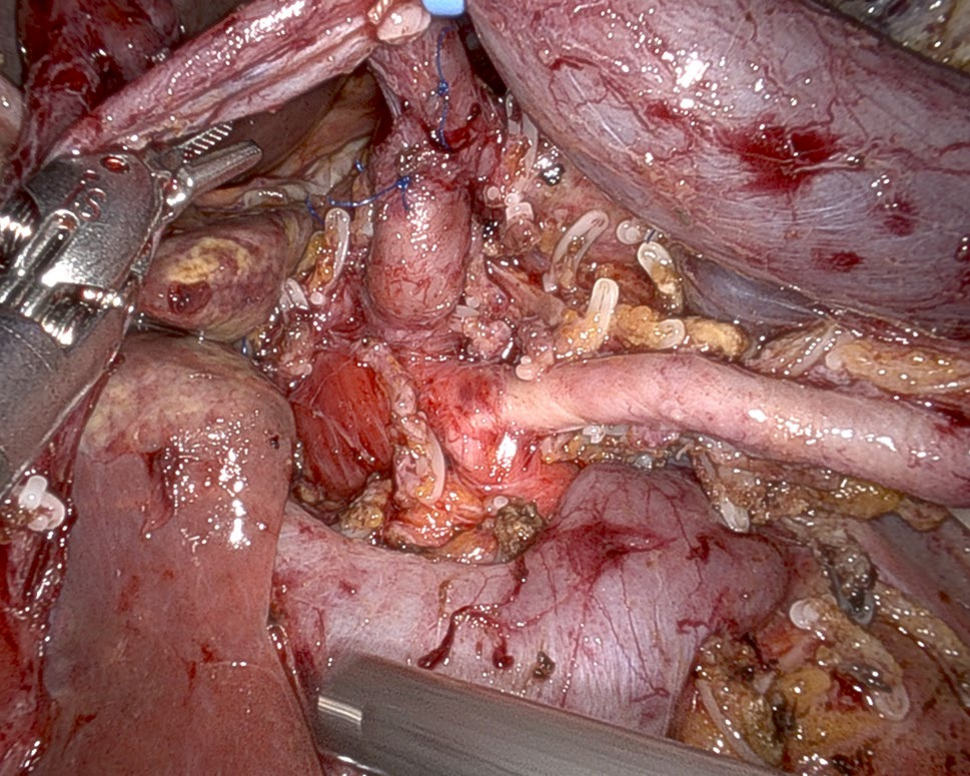
Completed triangle clearance of retroperitoneal lympho-neural tissues

While in most patients the SMA is approached from the right side, depending on individual tumor characteristics and patient’s anatomy, other routes may be preferred and all are possible working under robotic assistance [32]. In all patients, pancreaticoduodenal arteries are selectively identified and divided between ligatures or clips (Fig. 6). Dissection further proceeds proximally by peeling off periarterial tissues (Fig. 7) until dissection reaches the posterior area of centripetal lympho-neurectomy. At the end, the triangle space is totally cleared (Fig. 8) (video 4).

## Results

During the study period, a total of 252 RPDs were performed, including 127 (50.4%) C-Tr-RPDs for either PDAC (114; 89.8%) or malignant IPMN (13; 10.2%). Baseline characteristics of the 127 C-Tr-RPDs examined in this study are reported in Table 1.

**Table 1:** Baseline characteristics

Patients, *n*	127
Age, median (IQR)	67 (59–74)
Male gender, *n* (%)	50 (39.4%)
BMI, median (IQR), Kg/m^2^	24.6 (22–27)
ASA class, median (IQR)	3 (2–3)
Previous abdominal surgery, *n* (%)	68 (53.5%)
Neoadjuvant chemotherapy or chemoradiation therapy, *n* (%)	3 (2.3%)
Arterial variations in liver supply, *N*° (%)	15 (11.8%)
Right hepatic artery from SMA	9 (7.1%)
Common hepatic artery from SMA	2 (1.6%)
Accessory hepatic artery from SMA	2 (1.6%)
Artery for segment 6 from GDA	1 (0.8%)
Right hepatic artery arising from aorta	1 (0.8%)

*ASA* American Society of Anesthesiologists, *GDA* gastroduodenal artery, *SMA* superior mesenteric artery

### Operative outcome measures

Triangle dissection was carried out in all patients by cold dissection. Conversion to open PD was required in 3 patients (2.4%). Two conversions were required due to pneumoperitoneum intolerance. One additional patient needed to be converted to open PD during digestive reconstruction due to a short mesentery preventing tension-free approximation of jejunum to both pancreatic remnant and hepatic duct. Therefore, the composite primary endpoint of this study was not met in any of the 127 consecutive C-Tr-RPDs.

Other relevant intraoperative results are presented in Table 2. It may be worth to note that the vast majority of our patients underwent pylorus preservation (113; 88.9%) and that an associated vascular resection was required in 34 procedures (26.7%).

**Table 2:** Operative outcome measures

Conversion, *n* (%)	3 (2.4%)
Operative time, median (IQR)	540 (470–585)
Pylorus preserving, *n* (%)	113 (88.9%)
Vascular resection, *n* (%)	34 (26.7%)
Vein	30 (23.6%)
Artery	3 (2.3%)
Artery and vein	1 (0.8%)
Type of vein resection, *N*° (%)	
Type 1	1 (0.8%)
Type 2	12 (9.4%)
Type 3	11 (8.6%)
Type 4	7 (5.5%)
Intraoperative blood transfusion, median (IQR)	0 (0–1)

### Postoperative results

Postoperative results are reported in detail in Table 3. Postoperative mortality at 90 days was 7.1%. When the first 33 procedures were excluded, corresponding to our published learning curve [24], 90-day mortality dropped to 2.3%. Reasons for mortality during the learning curve were sepsis (*n* = 1), in the absence of intrabdominal complications, and delayed arterial bleeding (*n* = 2).

**Table 3:** Postoperative results

Length of hospital stay, median (IQR), days	17 (12–24)
90-day hospital readmission, *n* (%)	4 (3.1%)
90-day mortality, *n* (%)	9 (7.1%)
90-day mortality, after completion of the learning curve, *n* (%)	5 (2.3%)
Postoperative pancreatic fistula, *n* (%)	25 (22.0%)
Biochemical leak	11 (8.7%)
Grade B	10 (7.8%)
Grade C	4 (3.1%)
Delayed gastric emptying, *n* (%)	52 (40.9%)
Grade A	18 (14.2%)
Grade B	21 (16.5%)
Grade C	13 (10.2%)
Postpancreatectomy hemorrhage, *n* (%)	12 (9.4%)
Grade A	1 (0.8%)
Grade B	2 (1.6%)
Grade C	9 (7%)
Extraluminal	11 (8.6%)
Intraluminal	1 (0.8%)
Chyle leak, *n* (%)	4 (3.2%)
Postoperative complications, *n* (%)	
Clavien 0	34 (26.8%)
Clavien I	20 (15.8%)
Clavien II	47 (37.0%)
Clavien IIIa	8 (6.3%)
Clavien IIIb	7 (5.5%)
Clavien Iva	2 (1.5%)
Clavien IVb	0
Clavien V	
CCI, median (IQR)	20.9 (0–36.2)
Interventional procedures, *n* (%)	14 (11.0%)
Endovascular	3 (2.4%)
Percutaneous catheter drainage	11 (8.7%)
Reoperation, *n* (%)	14 (11.0%)

Postoperative complications developed in 94 patients (74.0%) and were not severe in 66 (51.9%). Overall, 99 patients (77.9%) had no or mild complications (grade I-II), while 28 (22.0%) had severe complications (≥ grade III). DGE was the most common postoperative complication (52; 40.9%), in the setting of a high rate of pylorus preservation. Median CCI was 20.9 (0–36.2).

We recorded no pseudoaneurysm of the gastroduodenal artery or any other retroperitoneal artery. One patient, operated in the early years of this experience, bled from the naked origin of the SMA. At the time of repeat surgery there was no evidence of pseudoaneurysm or local fluid collections containing pancreatic juice or bile. A clear explanation for this bleeding episode could not be demonstrated.

Fourteen patients (9.4%) required a reoperation, including 11 (8.6%) for extraluminal bleeding. The remaining indications for repeat surgery were intestinal volvulus (1; 0.8%) and fluid collections not amenable to percutaneous drainage (2; 1.6%). Considering that we have a low threshold for reoperation in the setting of extraluminal bleeding, to permit also removal of retroperitoneal hematoma, 6 cases (55.4%) were approached laparoscopically and hemorrhage was fixed through this approach in 4 patients (66.7%). Fluid collections requiring percutaneous catheter drainage were observed in 7 patients (9.7%).

### Pathology results

A summary of pathology results is presented in Table 4. A margin-negative resection was achieved in 71 patients (55.9%) in the context of high prevalence of perineural infiltration (109; 85.8%), a T3 rate of 41.7%, and an N2 rate of 36.2%. Among the 56 patients with positive margins (44.1%), R1 status was determined by the anterior margin in 5 patients (3.9%). Considering that the anterior margin is an anatomic margin, because no resection is carried out anteriorly, positive resection margins were seen in 51 patients (40.1%).

**Table 4:** Pathology results

PDAC, *n* (%)	114 (89.8%)
Malignant IPMN, *n* (%)	13 (10.2%)
Tumor size, median (IQR), mm	28 (25–55)
T status, *n* (%)	
T1	16 (12.6%)
T2	57 (44.9%)
T3	53 (41.7%)
T4	1 (0.8%)
N status, *n* (%)	
N0	21 (16.5%)
N1	60 (47.2%)
N2	46 (36.2%)
Margin status, *n* (%)	
R0	71 (55.9%)
R1	56 (44.1%)
R1 SMV margin	28 (50.0%)
R1 posterior margin	20 (35.7%)
R1 anterior margin	15 (26.8%)
R1 SMA margin	13 (23.2%)
R1 pancreatic neck margin	0
R1 common bile duct margin	0
R1 proximal duodenum/stomach	0
R1 at multiple margins	19 (33.9%)
Perineural infiltration	109 (85.8%)
Number of examined lymph nodes, median (IQR)	42 (33–51)
Number of metastatic lymph nodes, median (IQR)	4 (1–7)

*IPMN* intraductal papillary mucinous tumor, *mm* millimeters, *PDAC* pancreatic ductal adenocarcinoma, *SMA* superior mesenteric artery, *SMV* superior mesenteric vein

No patient was found to be R1 at the pancreatic neck margin, the common bile duct margin, and the proximal duodenum/stomach margin. However, based on frozen section histology, revision of pancreatic neck margin was required in 8 patients (6.3%).

In patients with positive resection margins, the most common site of R1 was the superior mesenteric vein margin (50.0%), followed by the posterior margin (35.7%) and the SMA margin (23.2%). In 19 patients, (33.9%) multiple margins were involved. In patients with hepatic arteries from the SMA, R1 resection rate was 33.3%.

## Discussion

Radical surgery performed at the price of a reasonable rate of postoperative complications is the main surgeon contribution to the cure of patients diagnosed with PDAC.

In open PD, triangle dissection improves the number of examined lymph nodes and decreases the rate of (direct) margin positivity, without increasing postoperative complications and mortality [33]. The technique of triangle RPD was recently reported both by Kinny‐Köster [34] and Machado [35]. Perivascular dissection was carried out using harmonic shears and robotic hook, respectively. Outcomes of triangle RPD were described only by Machado. In 22 procedures performed for unspecified tumor types, there were 6 vein resections (27.2%) and one R1 resection (4.5%). The mean number of examined lymph nodes was 40 (range: 27–77). Median time from surgery to chemotherapy was 23 days. There were no postoperative deaths, one patient developed chyle leak, and an additional patient suffered from diarrhea (resolved after 3 months) [35]. Shyr reported on 36 RPDs with level 3 mesopancreas dissection, a procedure that corresponds to triangle RPD. When compared to RPD with less extended mesopancreas dissection (i.e., level 1 and level 2), triangle RPD was associated with longer median operation time and higher blood loss, but equivalent incidence and severity of postoperative complications, higher R0 rates, and increased number of examined lymph nodes [14]. No detail was provided on technique of periadvential artery dissection (i.e., sharp or energized).

In this article we have shown the feasibility of C-Tr-RPDs. The composite primary endpoint of this study (conversion to open surgery due to inability to complete triangle dissection or need to use energy devices) was never met in 127 consecutive procedures. Despite our series includes also a good proportion of RPDs with associated vascular procedures, feasibility of C-Tr-RPD does not mean that this procedure could be performed in an unselected group of patients with PDAC.

Clearly, our experience started years before the triangle concept was proposed by Adham [7]. The rationale for triangle (lympho-)neurectomy is provided by the anatomy of ExNP and the proclivity of PDAC to spread along nerve sheaths [8, 9]. Considering that extrapancreatic plexus invasion occurs in 72–79% of PADC located in the head/uncinated process of the pancreas [9, 36], leaving behind triangle tissues seems to prepare the ground for local recurrence in some patients.

We have reported a quite high mortality rate at 90 days (7.1%) that could rise concerns on safety of C-Tr-RPD. However, 90-day mortality decreased to 2.3% when the analysis was restricted to the patients operated after completion of the learning curve [24]. In addition, RPD, alike open PD [37], is not a uniform procedure. In our series a vascular resection was required in 26.7% of C-Tr-RPDs, and arterial variations in liver supply were recorded in 11.8% of the patients. At a difference from most other reports on RPD with vascular resection [35, 38, 39], our vein resections were mostly segmental resections (type 3: 11; 35.5%–type 4: 7; 22.5%). While segmental vein resection perfectly matches the oncologic principles of triangle PD, it is clear that it increases technical complexity. Of the 50 RPDs with associated vein resection reported by the Pittsburgh group, only one patient had a segmental vein resection. In this series, 90-day mortality was 8.0% [38]. Machado described 6 cases of vein resection during RPD, without details on the type of vein resection [35]. Giulianotti reported on 6 vascular resections during robotic pancreatectomy, including 2 RPDs with side-wall vein resection [39]. Marino described 10 RPDs with vein resection, including 5 type 3 and 2 type 4 resections. In this series, RPD with vein resection, when compared to standard RPD, was associated with longer operative time, increased blood loss, and higher need of blood transfusions. Although conversion to open (10.0%) and 90-day mortality (10.0%) did not reach statistical significance, possibly because or small sample size, RPD with vein resection was associated with high conversion and mortality rates [40].

We believe that cold dissection could be important to avoid collateral energy damage to visceral arteries. In this respect it is important to note that we recorded no pseudoaneurysm of the gastroduodenal artery (PSA-GDA). In many articles reporting on RPD there is no detailed description of PSA-GDA. The Pittsburgh group in 500 RPDs, employing a radiofrequency dissection device [41], reported a 4.2% incidence of PSA-GDA that did not decreased in the last 100 procedures (5.0%) [42]. In a systematic review of the literature, Brodie showed that PSA-GDA occurred in 55 patients following 4227 pancreatic procedures (1.3%). Incidence of PSA-GDA in individual studies ranged between 0.2% and 8.3% [43]. PSA-GDA typically develops in patients with clinically relevant postoperative pancreatic fistula or other local complications causing vascular erosion. However, in up to 25% of the patients with major arterial bleeding following PD, pseudoaneurysms arise in the absence of erosive factors and far from the gastroduodenal artery stump possibly because of minor iatrogenic injuries overlooked at the time of surgery [44–46]. No proof exists that the use of modern energy devices near the wall of large visceral arteries increases the risk of nonerosive pseudoaneurysm, but experimental evidence shows that these instruments produce lateral thermal spread [47] that, at least in theory, could damage the fragile wall of visceral arteries. Additionally, sparing the use of energy could reduce both costs [48] and CO2 emissions [49]. Reduction of carbon footprint of energy devices is expected to contribute to the implementation of “green operating rooms” [50, 51].

Extensive clearance of triangle tissues could increase the rate of chyle leak. A recent systematic review of the literature showed that PD is associated with a chyle leak rate of 6.6% [52]. In our series, careful clipping of large lymphatics resulted in a chyle leak rate of 3.2% despite extensive clearance of retroperitoneal tissues. Chyle leaks had a benign clinical course and resolved with dietary interventions and medical therapy.

Our data underscore the importance of the learning curve of robotic PD. A national Dutch trial showed that a combination of video review, simulation exercises, biotissue drills, and on-site proctoring permits safe implementation of robotic PD, in centers with sufficient surgical volume [53]. Currently, most surgeons used energized dissection to prevent bleeding from small vessels and speed up the procedure. Therefore, adding the concept of cold perivascular dissection could further complicate the learning process of robotic PD. We suggest that cold perivascular dissection should be first practiced in the open setting and progressively implemented in robotic PD.

Our pathology data confirm that triangle PD increases lymph node retrieval [33]. However, we have reported a quite high rate of R1 resections (44.1%). It is worth to note that 15 of our patients (11.8%) were R1 at the anterior margin (11.8%), where no resection is performed. In addition, 28 patients had a positive vein bed margin. The vein bed margin is the most affected margin following PD for PDAC [54]. In 1998, Ishiwawa demonstrated that cancer cells can be detected on the wall of 30% of seemingly noninvolved veins following PD by intraoperative cytology. Once these veins were resected, tumor involvement was confirmed in 86% of the specimens [55]. Finally, 19 of our patients (14.9%) had multiple positive margins. These figures cannot be justified by “limited” surgery, as shown by the number of examined lymph nodes and our videos. They rather underscore the importance of excellent pathology to clearly depict the local status of resected PDAC. Our data are indeed in agreement with studies on resection margins by prominent pancreatic pathologists [25, 56].

This study has some limitations. First, most surgeons use energy devices for dissection in both open and minimally invasive PD without a clear increase in the rate of pseudoaneurysm. Our favorable experience and familiarity with cold perivascular dissection in open PD prompted us to implement this technique also in RPD. Therefore, diffusion of C-Tr-RPD could be difficult. Second, this series includes only few patients who received neoadjuvant treatments. Sclerosis associated with these treatments could complicate performance of cold triangle dissection.

In conclusion, C-Tr-RPD is feasible, carries a risk of surgical complications commensurate to the magnitude of the procedure, and improves staging of resected PDAC. Although not mandatory, demonstration of feasibility of triangle dissection without the use of energy devices sheds further light on the potential of robotic assistance in minimally invasive PD.

## Supplementary Information

Below is the link to the electronic supplementary material.

Supplementary file1 (MPG 146602 KB)

Supplementary file2 (MPG 95824 KB)

Supplementary file3 (MPG 77408 KB)

Supplementary file4 (MPG 213134 KB)

## References

[1] Rhim AD, Mirek ET, Aiello NM, Maitra A, Bailey JM, McAllister F, Reichert M, Beatty GL, Rustgi AK, Vonderheide RH, Leach SD, Stanger BZ (2012). EMT and dissemination precede pancreatic tumor formation. Cell.

[2] Dillhoff M, Pawlik TM (2021). Role of node dissection in pancreatic tumor resection. Ann Surg Oncol.

[3] Schwarz RE, Smith DD (2006). Extent of lymph node retrieval and pancreatic cancer survival: information from a large US population database. Ann Surg Oncol.

[4] Mirkin KA, Hollenbeak CS, Wong J (2017). Greater lymph node retrieval and lymph node ratio impacts survival in resected pancreatic cancer. J Surg Res.

[5] Kaltenmeier C, Nassour I, Hoehn RS, Khan S, Althans A, Geller DA, Paniccia A, Zureikat A, Tohme S (2021). Impact of resection margin status in patients with pancreatic cancer: a national cohort study. J Gastrointest Surg.

[6] Sandini M, Ruscic KJ, Ferrone CR, Qadan M, Eikermann M, Warshaw AL, Lillemoe KD, Castillo CF (2019). Major complications independently increase long-term mortality after pancreatoduodenectomy for cancer. J Gastrointest Surg.

[7] Adham M, Singhirunnusorn J (2012). Surgical technique and results of total mesopancreas excision (TMpE) in pancreatic tumors. Eur J Surg Oncol.

[8] Nagakawa T, Kayahara M, Ohta T, Ueno K, Konishi I, Miyazaki I (1991). Patterns of neural and plexus invasion of human pancreatic cancer and experimental cancer. Int J Pancreatol.

[9] Nagakawa T, Kayahara M, Ueno K, Ohta T, Konishi I, Ueda N, Miyazaki I (1992). A clinicopathologic study on neural invasion in cancer of the pancreatic head. Cancer.

[10] Schneider M, Strobel O, Hackert T, Büchler MW (2019). Pancreatic resection for cancer-the Heidelberg technique. Langenbecks Arch Surg.

[11] Groot VP, Rezaee N, Wu W, Cameron JL, Fishman EK, Hruban RH, Weiss MJ, Zheng L, Wolfgang CL, He J (2018). Patterns, timing, and predictors of recurrence following pancreatectomy for pancreatic ductal adenocarcinoma. Ann Surg.

[12] Heye T, Zausig N, Klauss M, Singer R, Werner J, Richter GM, Kauczor HU, Grenacher L (2011). CT diagnosis of recurrence after pancreatic cancer: is there a pattern?. World J Gastroenterol.

[13] Boggi U, Del Chiaro M, Croce C, Vistoli F, Signori S, Moretto C, Amorese G, Mazzeo S, Cappelli C, Campani D, Mosca F (2009). Prognostic implications of tumor invasion or adhesion to peripancreatic vessels in resected pancreatic cancer. Surgery.

[14] Shyr BU, Shyr BS, Chen SC, Shyr YM, Wang SE (2021). Mesopancreas level 3 dissection in robotic pancreaticoduodenectomy. Surgery.

[15] Cuschieri A (2005). Reducing errors in the operating room: surgical proficiency and quality assurance of execution. Surg Endosc.

[16] World Medical Association (2013). World medical association declaration of Helsinki: ethical principles for medical research involving human subjects. JAMA.

[17] von Elm E, Altman DG, Egger M, Pocock SJ, Gøtzsche PC, Vandenbroucke JP, Initiative STROBE (2014). The Strengthening the Reporting of Observational Studies in Epidemiology (STROBE) Statement: guidelines for reporting observational studies. Int J Surg.

[18] Dindo D, Demartines N, Clavien PA (2004). Classification of surgical complications: a new proposal with evaluation in a cohort of 6336 patients and results of a survey. Ann Surg.

[19] Wente MN, Veit JA, Bassi C, Dervenis C, Fingerhut A, Gouma DJ, Izbicki JR, Neoptolemos JP, Padbury RT, Sarr MG, Yeo CJ, Büchler MW (2007). Postpancreatectomy hemorrhage (PPH): an International Study Group of Pancreatic Surgery (ISGPS) definition. Surgery.

[20] Wente MN, Bassi C, Dervenis C, Fingerhut A, Gouma DJ, Izbicki JR, Neoptolemos JP, Padbury RT, Sarr MG, Traverso LW, Yeo CJ, Büchler MW (2007). Delayed gastric emptying (DGE) after pancreatic surgery: a suggested definition by the International Study Group of Pancreatic Surgery (ISGPS). Surgery.

[21] Besselink MG, van Rijssen LB, Bassi C, Dervenis C, Montorsi M, Adham M, Asbun HJ, Bockhorn M, Strobel O, Büchler MW, Busch OR, Charnley RM, Conlon KC, Fernández-Cruz L, Fingerhut A, Friess H, Izbicki JR, Lillemoe KD, Neoptolemos JP, Sarr MG, Shrikhande SV, Sitarz R, Vollmer CM, Yeo CJ, Hartwig W, Wolfgang CL, Gouma DJ, International Study Group on Pancreatic Surgery (2017). Definition and classification of chyle leak after pancreatic operation: a consensus statement by the international study group on pancreatic surgery. Surgery.

[22] Slankamenac K, Graf R, Barkun J, Puhan MA, Clavien PA (2013). The comprehensive complication index: a novel continuous scale to measure surgical morbidity. Ann Surg.

[23] Bockhorn M, Uzunoglu FG, Adham M, Imrie C, Milicevic M, Sandberg AA, Asbun HJ, Bassi C, Büchler M, Charnley RM, Conlon K, Cruz LF, Dervenis C, Fingerhutt A, Friess H, Gouma DJ, Hartwig W, Lillemoe KD, Montorsi M, Neoptolemos JP, Shrikhande SV, Takaori K, Traverso W, Vashist YK, Vollmer C, Yeo CJ, Izbicki JR, International Study Group of Pancreatic Surgery (2014). Borderline resectable pancreatic cancer: a consensus statement by the International Study Group of Pancreatic Surgery (ISGPS). Surgery.

[24] Napoli N, Kauffmann EF, Palmeri M, Miccoli M, Costa F, Vistoli F, Amorese G, Boggi U (2016). The learning curve in robotic pancreaticoduodenectomy. Dig Surg.

[25] Verbeke CS, Leitch D, Menon KV, McMahon MJ, Guillou PJ, Anthoney A (2006). Redefining the R1 resection in pancreatic cancer. Br J Surg.

[26] Kauffmann EF, Napoli N, Menonna F, Vistoli F, Amorese G, Campani D, Pollina LE, Funel N, Cappelli C, Caramella D, Boggi U (2016). Robotic pancreatoduodenectomy with vascular resection. Langenbecks Arch Surg.

[27] Boggi U, Signori S, De Lio N, Perrone VG, Vistoli F, Belluomini M, Cappelli C, Amorese G, Mosca F (2013). Feasibility of robotic pancreaticoduodenectomy. Br J Surg.

[28] Napoli N, Kauffmann EF, Menonna F, Perrone VG, Brozzetti S, Boggi U (2016). Indications, technique, and results of robotic pancreatoduodenectomy. Updates Surg.

[29] Kauffmann EF, Napoli N, Cacace C, Menonna F, Vistoli F, Amorese G, Boggi U (2020). Resection or repair of large peripancreatic arteries during robotic pancreatectomy. Updates Surg.

[30] Nagakawa Y, Nakata K, Nishino H, Ohtsuka T, Ban D, Asbun HJ, Boggi U, He J, Kendrick ML, Palanivelu C, Liu R, Wang SE, Tang CN, Takaori K, Abu Hilal M, Goh BKP, Honda G, Jang JY, Kang CM, Kooby DA, Nakamura Y, Shrikhande SV, Wolfgang CL, Yiengpruksawan A, Yoon YS, Watanabe Y, Kozono S, Ciria R, Berardi G, Garbarino GM, Higuchi R, Ikenaga N, Ishikawa Y, Maekawa A, Murase Y, Zimmitti G, Kunzler F, Wang ZZ, Sakuma L, Takishita C, Osakabe H, Endo I, Tanaka M, Yamaue H, Tanabe M, Wakabayashi G, Tsuchida A, Nakamura M (2021). International expert consensus on precision anatomy for minimally invasive pancreatoduodenectomy: PAM-HBP surgery project. J Hepatobiliary Pancreat Sci.

[31] Nakata K, Higuchi R, Ikenaga N, Sakuma L, Ban D, Nagakawa Y, Ohtsuka T, Asbun HJ, Boggi U, Tang CN, Wolfgang CL, Nishino H, Endo I, Tsuchida A, Nakamura M, Study Group of Precision Anatomy for Minimally Invasive Hepato-Biliary-Pancreatic surgery (PAM-HBP Surgery) (2021). Precision anatomy for safe approach to pancreatoduodenectomy for both open and minimally invasive procedure: a systematic review. J Hepatobiliary Pancreat Sci.

[32] Sanjay P, Takaori K, Govil S, Shrikhande SV, Windsor JA (2012). 'Artery-first' approaches to pancreatoduodenectomy. Br J Surg.

[33] Klotz R, Hackert T, Heger P, Probst P, Hinz U, Loos M, Berchtold C, Mehrabi A, Schneider M, Müller-Stich BP, Strobel O, Diener MK, Mihaljevic AL, Büchler MW (2021). The TRIANGLE operation for pancreatic head and body cancers: early postoperative outcomes. HPB (Oxford).

[34] Kinny-Köster B, Habib JR, Javed AA, Shoucair S, van Oosten AF, Fishman EK, Lafaro KJ, Wolfgang CL, Hackert T, He J (2021). Technical progress in robotic pancreatoduodenectomy: TRIANGLE and periadventitial dissection for retropancreatic nerve plexus resection. Langenbecks Arch Surg.

[35] Machado MA, Mattos BV, Lobo Filho MM, Makdissi F (2021). Mesopancreas excision and triangle operation during robotic pancreatoduodenectomy. Ann Surg Oncol.

[36] Makino I, Kitagawa H, Ohta T, Nakagawara H, Tajima H, Ohnishi I, Takamura H, Tani T, Kayahara M (2008). Nerve plexus invasion in pancreatic cancer: spread patterns on histopathologic and embryological analyses. Pancreas.

[37] Mihaljevic AL, Hackert T, Loos M, Hinz U, Schneider M, Mehrabi A, Hoffmann K, Berchtold C, Müller-Stich BP, Diener M, Strobel O, Büchler MW (2021). Not all Whipple procedures are equal: proposal for a classification of pancreatoduodenectomies. Surgery.

[38] Beane JD, Zenati M, Hamad A, Hogg ME, Zeh HJ, Zureikat AH (2019). Robotic pancreatoduodenectomy with vascular resection: Outcomes and learning curve. Surgery.

[39] Giulianotti PC, Addeo P, Buchs NC, Ayloo SM, Bianco FM (2011). Robotic extended pancreatectomy with vascular resection for locally advanced pancreatic tumors. Pancreas.

[40] Marino MV, Giovinazzo F, Podda M, Gomez Ruiz M, Gomez Fleitas M, Pisanu A, Latteri MA, Takaori K (2020). Robotic-assisted pancreaticoduodenectomy with vascular resection. Description of the surgical technique and analysis of early outcomes. Surg Oncol.

[41] Nguyen KT, Zureikat AH, Chalikonda S, Bartlett DL, Moser AJ, Zeh HJ (2011). Technical aspects of robotic-assisted pancreaticoduodenectomy (RAPD). J Gastrointest Surg.

[42] Zureikat AH, Beane JD, Zenati MS, Al Abbas AI, Boone BA, Moser AJ, Bartlett DL, Hogg ME, Zeh HJ (2021). 500 Minimally invasive robotic pancreatoduodenectomies: one decade of optimizing performance. Ann Surg.

[43] Brodie B, Kocher HM (2019). Systematic review of the incidence, presentation and management of gastroduodenal artery pseudoaneurysm after pancreatic resection. BJS Open.

[44] Fujii Y, Shimada H, Endo I, Yoshida K, Matsuo K, Takeda K, Ueda M, Morioka D, Tanaka K, Togo S (2007). Management of massive arterial hemorrhage after pancreatobiliary surgery: does embolotherapy contribute to successful outcome?. J Gastrointest Surg.

[45] Sugimoto H, Kaneko T, Ishiguchi T, Takai K, Ohta T, Yagi Y, Inoue S, Takeda S, Nakao A (2001). Delayed rupture of a pseudoaneurysm following pancreatoduodenectomy: report of a case. Surg Today.

[46] Feng F, Cao X, Liu X, Qin J, Xing Z, Duan J, Liu C, Liu J (2019). Two forms of one complication: late erosive and nonerosive postpancreatectomy hemorrhage following laparoscopic pancreaticoduodenectomy. Medicine (Baltimore).

[47] Emam TA, Cuschieri A (2003). How safe is high-power ultrasonic dissection?. Ann Surg.

[48] Manatakis DK, Georgopoulos N (2014). Reducing the cost of laparoscopy: reusable versus disposable laparoscopic instruments. Minim Invasive Surg.

[49] Ibbotson S, Dettmer T, Kara S (2013). Eco-efficiency of disposable and reusable surgical instruments—a scissors case. Int J Life Cycle Assess.

[50] Rizan C, Steinbach I, Nicholson R, Lillywhite R, Reed M, Bhutta MF (2020). The carbon footprint of surgical operations: a systematic review. Ann Surg.

[51] Anand SK, Culver LG, Maroon J (2022). Green operating room-current standards and insights from a large North American medical center. JAMA Surg.

[52] Varghese C, Wells CI, Lee S, Pathak S, Siriwardena AK, Pandanaboyana S (2001). Systematic review of the incidence and risk factors for chyle leak after pancreatic surgery. Surgery.

[53] Zwart MJW, Nota CLM, de Rooij T (2021). Outcomes of a multicenter training program in robotic pancreatoduodenectomy (LAELAPS-3. Ann Surg.

[54] Groen JV, van Manen L, van Roessel S, van Dam JL, Bonsing BA, Doukas M, van Eijck CHJ, Farina Sarasqueta A, Putter H, Vahrmeijer AL, Verheij J, Besselink MG, Groot Koerkamp B, Mieog JSD (2001). Resection of the portal-superior mesenteric vein in pancreatic cancer: pathological assessment and recurrence patterns. Pancreas.

[55] Ishikawa O, Ohigashi H, Sasaki Y, Nakano H, Furukawa H, Imaoka S, Takenaka A, Kasugai T, Ishiguro S (1998). Intraoperative cytodiagnosis for detecting a minute invasion of the portal vein during pancreatoduodenectomy for adenocarcinoma of the pancreatic head. Am J Surg.

[56] Esposito I, Kleeff J, Bergmann F, Reiser C, Herpel E, Friess H, Schirmacher P, Büchler MW (2008). Most pancreatic cancer resections are R1 resections. Ann Surg Oncol.

